# CVOT Summit 2022 Report: new cardiovascular, kidney, and glycemic outcomes

**DOI:** 10.1186/s12933-023-01788-6

**Published:** 2023-03-16

**Authors:** Oliver Schnell, Tadej Battelino, Richard Bergenstal, Andreas L. Birkenfeld, Antonio Ceriello, Alice Cheng, Melanie Davies, Steve Edelman, Thomas Forst, Francesco Giorgino, Jennifer Green, Per-Henrik Groop, Samy Hadjadj, Hiddo J.L.Heerspink, Marcus Hompesch, Baruch Izthak, Linong Ji, Naresh Kanumilli, Boris Mankovsky, Chantal Mathieu, Martin Miszon, Reem Mustafa, Michael Nauck, Roberto Pecoits-Filho, Jeremy Pettus, Kari Ranta, Helena W. Rodbard, Peter Rossing, Lars Ryden, Petra-Maria Schumm-Draeger, Scott D. Solomon, Jan Škrha, Pinar Topsever, Tina Vilsbøll, John Wilding, Eberhard Standl

**Affiliations:** 1 Forschergruppe Diabetes e. V., Helmholtz Center Munich, Ingolstaedter Landstraße 1, Neuherberg, 85764 (Munich), Germany; 2 University Medical Center, Ljubljana, Slovenia; 3 University Children’s Hospital, Ljubljana, Slovenia; 4 Faculty of Medicine, University of Ljubljana, Ljubljana, Slovenia; 5 International Diabetes Center at Park Nicollet, Health Partners, Minneapolis, MN USA; 6 Department of Internal Medicine IV, University Clinic Tübingen, Tübingen, Germany; 7 Institute for Diabetes Research and Metabolic Diseases at the Eberhard-Karls-University of Tübingen, Tübingen, Germany; 8 German Center for Diabetes Research (DZD e.V.), Tübingen, Germany; 9 IRCCS MultiMedica, Milan, Italy; 10 Credit Valley Hospital, Mississauga, ON Canada; 11 Diabetes Research Centre, University of Leicester, Leicester, UK; 12 NIHR Biomedical Research Centre, Leicester, UK; 13 Taking Control of Your Diabetes, Solana Beach, CA USA; 14 CRS Clinical Research Services Mannheim GmbH, Mannheim, Germany; 15 Department of Precision and Regenerative Medicine and Ionian Area, University of Bari Aldo Moro, Bari, Italy; 16 Division of Endocrinology, Department of Medicine and Duke Clinical Research Institute, Duke University School of Medicine, Durham, NC USA; 17 Department of Nephrology, University of Helsinki and Helsinki University Hospital, Helsinki, Finland; 18 Folkhälsan Institute of Genetics, Folkhälsan Research Center, Biomedicum Helsinki, University of Helsinki, Helsinki, Finland; 19 Department of Diabetes, Central Medical School, Monash University, Melbourne, Australia; 20 Thorax Institute, University Hospital of Nantes, Nantes, France; 21 Department of Clinical Pharmacy and Pharmacology, University Medical Center Groningen, University of Groningen, Groningen, The Netherlands; 22 ProSciento Inc., San Diego, CA USA; 23 Clalit Health Services and Technion Faculty of Medicine, Haifa, Israel; 24 Peking University People’s Hospital, Xicheng District, Beijing, China; 25 Manchester University Foundation Trust, Manchester, UK; 26 Shupyk National Medical Academy of Postgraduate Education, Kiev, Ukraine; 27 Department of Endocrinology, Catholic University Leuven, Leuven, Belgium; 28 Sciarc GmbH, Baierbrunn, Germany; 29 Division of Nephrology and Hypertension, Medical Center, University of Kansas, Kansas City, KS USA; 30 Diabetes Division, St. Josef Hospital, Ruhr-University Bochum, Bochum, Germany; 31 Pontifical Catholic University of Paraná, Curitiba, Brazil; 32 Altman Clinical and Translational Research Institute (ACTRI), La Jolla, CA USA; 33 Eli Lilly and Company, Indianapolis, IN USA; 34 Endocrine and Metabolic Consultants, Rockville, MD USA; 35 Steno Diabetes Center Copenhagen, Herlev, Denmark; 36 Department of Clinical Medicine, University of Copenhagen, Copenhagen, Denmark; 37 Department of Medicine K2, Karolinska Institute, Stockholm, Sweden; 38 Center Internal Medicine Fünf Höfe, Munich, Germany; 39 Cardiovascular division, Brigham and Women’s Hospital, Boston, MA USA; 40 Third Medical Department and Laboratory for Endocrinology and Metabolism, First Faculty of Medicine, Charles University, Prague, Czech Republic; 41 Department of Family Medicine, Acıbadem Mehmet Ali Aydınlar University School of Medicine, Istanbul, Turkey; 42 Center for Diabetes Research, Gentofte Hospital, University of Copenhagen, Hellerup, Denmark; 43 Institute of Life Course and Medical Sciences, University of Liverpool, Liverpool, UK

**Keywords:** Cardiovascular disease, Chronic kidney disease, Diabetes, GIP/GLP-1 receptor agonist, Heart failure, Obesity, SGLT2 inhibitor

## Abstract

The 8th Cardiovascular Outcome Trial (CVOT) Summit on Cardiovascular, Kidney, and Glycemic Outcomes was held virtually on November 10–12, 2022. Following the tradition of previous summits, this reference congress served as a platform for in-depth discussion and exchange on recently completed outcomes trials as well as key trials important to the cardiovascular (CV) field. This year’s focus was on the results of the DELIVER, EMPA-KIDNEY and SURMOUNT-1 trials and their implications for the treatment of heart failure (HF) and chronic kidney disease (CKD) with sodium-glucose cotransporter-2 (SGLT2) inhibitors and obesity with glucose-dependent insulinotropic polypeptide (GIP) and glucagon-like peptide-1 (GLP-1) receptor agonists. A broad audience of primary care physicians, diabetologists, endocrinologists, cardiologists, and nephrologists participated online in discussions on new consensus recommendations and guideline updates on type 2 diabetes (T2D) and CKD management, overcoming clinical inertia, glycemic markers, continuous glucose monitoring (CGM), novel insulin preparations, combination therapy, and reclassification of T2D. The impact of cardiovascular outcomes on the design of non-alcoholic fatty liver disease (NAFLD) and non-alcoholic steatohepatitis (NASH) trials, as well as the impact of real-world evidence (RWE) studies on the confirmation of CVOT outcomes and clinical trial design, were also intensively discussed. The 9th Cardiovascular Outcome Trial Summit will be held virtually on November 23–24, 2023 (http://www.cvot.org).

## Background

The prevalence of diabetes mellitus continues to rise and is reaching alarming levels. The International Diabetes Federation (IDF) estimates that the number of people with diabetes will increase from 537 million (10.5%) in 2021 to 783.2 million (12.2%) in 2045 [1, 2]. Complications are frequent in diabetes. For example, cardiovascular disease (CVD) is diagnosed in more than 30% of individuals with type 2 diabetes (T2D) [3, 4]. Similarly, at least 40% of people with T2D develop diabetic kidney disease (DKD), which is a major cause of chronic kidney disease (CKD) [5, 6]. Besides cancer, CVD, CKD, and diabetes are the leading causes of death worldwide. CVD-related deaths have increased by more than 25% and CKD- and diabetes-related deaths have nearly doubled since 1990 [6]. CVD is considered the leading cause of mortality and morbidity in those with T2D, and life expectancy is estimated to be reduced by 16 years in individuals with T2D and diagnosed CKD [7].

The continued development of effective, accessible, affordable, and safe pharmacological agents is necessary to minimize the health-damaging complications of diabetes. In 2008, it became mandatory to evaluate all new T2D therapies in long-term CV outcomes trials (CVOTs) [8, 9]. Until 2021, 21 CVOTs have been conducted, primarily for three new glucose-lowering drug classes: glucagon-like peptide-1 receptor agonists (GLP-1 RAs) [10–15], dipeptidyl peptidase-4 inhibitors (DPP-4is) [16–20], and sodium-glucose cotransporter-2 inhibitors (SGLT2is), [21–26]; in addition to these substance classes, a novel mineralocorticoid receptor antagonist (MRA) has been investigated in individuals with CKD and T2D [27]. In 2022, the list of outcomes trials was expanded by two trials with SGLT2i: one in individuals with HF and preserved ejection fraction (HFpEF) with or without diabetes (DELIVER, with dapagliflozin) [28] and one evaluating the efficacy and safety on the progression of kidney disease and CVD in individuals with CKD (EMPA-KIDNEY, with empagliflozin) [29]. In addition, a study evaluating the effect of a novel glucose-dependent insulinotropic polypeptide (GIP) and GLP-1 RA on weight loss in people without T2D (SURMOUNT-1, with tirzepatide) was completed [30], paving for future planned CVOTs (NCT04255433).

Most CVOTs included the core composite outcome of 3-Point MACE (3P-MACE, consisting of CV death, nonfatal MI, and nonfatal stroke). The trials with SGLT2i and GLP-1 RA revealed that most of them had a beneficial impact on 3P-MACE, in contrast to trials with DPP-4i. Furthermore, many CVOTs have reported on outcomes beyond the required 3P-MACE, demonstrating additional benefits of some glucose-lowering drugs, such as reducing the risk of hospitalization for heart failure (HHF) and slowing the progression of kidney disease. For SGLT2is, improved kidney outcomes have been consistently observed in all CVOTs, both in terms of less albuminuria and preservation of kidney function [21–26]. In contrast, GLP-1 RA CVOTs showed improvements in outcomes related to albuminuria, although generally neutral effects were seen in other kidney outcomes [10–15].

However, dulaglutide demonstrated an improvement in the combined endpoint of albuminuria and deterioration of estimated glomerular filtration rate (eGFR) > 30% in the REWIND trial [31]. Furthermore, The AMPLITUDE-O CVOT study published in 2021 was the first GLP-1 RA CVOT study to demonstrate relative risk reductions (RRRs) of 32% for a composite kidney outcome, defined as progression to macroalbuminuria, ≥ 30% increase in urine albumin-to-creatinine ratio (UACR), sustained decrease in the eGFR of ≥ 40% for ≥ 30 days, or sustained eGFR of < 15 mL per minute per 1.73 m^2^ for ≥ 30 days and kidney replacement therapy for ≥ 90 days [15]. Furthermore, 15% of individuals were on SGLT2i at baseline, and post-hoc analysis showed similar benefit of GLP-1 with SGLT2i as in those on SGLT2i, with no suggestion of additive benefit [32].

Evidence from CVOTs has been incorporated into the recommendations of several international guidelines. For example, the general consensus of the American Diabetes Association (ADA), the American College of Cardiology (ACC), the European Association for the Study of Diabetes (EASD), and the European Society of Cardiology (ESC) is that individuals diagnosed with T2D and CVD should be treated with an SGLT2i or GLP-1 RA with proven CVD benefit, either as initial add-on therapy to metformin or as monotherapy [33–35].

As in previous years [36–41], we present and summarize key aspects discussed at the 8th CVOT Summit held virtually on November 10–11, 2022. The CVOT Summit on cardiovascular, kidney, and glycemic outcomes 2022, was an interdisciplinary platform, which was also organized in conjunction with five study groups: Primary Care Diabetes Europe (PCDE, www.pcdeurope.org), Diabetes and Cardiovascular Disease EASD Study Group (DCVD, www.dcvd.org), European Diabetic Nephropathy Study Group (EDNSG, www.ednsg.org) the European Incretin Study Group (www.increti-studygroup.ch) and the Working Group Diabetes & Herz (www.ddg.org). Participants from 55 countries and five continents with specialties in diabetology, endocrinology, cardiology, nephrology, and primary care contributed to the discussions of the CVOT Summit on Cardiovascular and Renal Outcomes 2022 (www.cvot.org).

## **Updates on CVOTs**

A summary of the characteristics and results of HF, CKD and CV outcome trials published in 2022 is listed in Tables 1, 2, 3.

**Table 1:** Key information of the DELIVER trial

DELIVER [28]		
Class & Cardiovascular (CV) outcomes	HR (95% CI)	p-value
**Primary composite outcome**		
Composite of worsening heart failure (HF) (defined as unplanned hospitalization for HF or an urgent visit for HF or CV death)	0.82 (0.73–0.92)	< 0.001
**Primary outcome**		
CV death	0.88 (0.74–1.05)	
**Secondary outcome**		
Total number of worsening HF events and CV deaths	0.77 (0.67–0.89)	< 0.001
**Secondary outcome**		
Change in KCCQ^a^ total symptom score at month 8	1.11 (1.03–1.21)	< 0.009
**Secondary outcome**		
Mean change in KCCQ^a^ total symptom score at month 8 among survivors	2.4 (1.5–3.4)	
**Secondary outcome**		
Death from any cause	0.94 (0.83–1.07)	

Adverse events	Event rate (%) active vs. placebo group	
Any serious adverse events (death included)	43.5 vs. 45.5	

^a^The Kansas City Cardiomyopathy Questionnaire (KCCQ) is a 23-item self-administered questionnaire developed to independently measure the participant’s perception of their health status, which includes heart failure symptoms, impact on physical and social function, and how their heart failure impacts their quality of life (QOL) within a 2 week recall period

**Table 2:** Key information of the EMPA-KIDNEY trial

EMPA-KIDNEY		
Class & Cardiovascular (CV) outcomes	HR (95% CI)	p-value
**Primary composite outcome**		
Progression of kidney disease or death from CV causes	0.72 (0.64–0.82)	< 0.001
**Key secondary outcome**		
Hospitalization for heart failure or death from CV causes	0.84 (0.67–1.07)	0.15
**Key secondary outcome**		
Hospitalization for any cause^a^	0.86 (0.78–0.95)	0.003
**Key secondary outcome**		
Death from any cause	0.87 (0.70–1.08)	0.21
**Secondary outcome**		
Progression of kidney disease	0.71 (0.62–0.81)	
**Secondary outcome**		
Death from CV causes	0.84 (0.60–1.19)	
**Secondary outcome**		
End-stage kidney disease or death from CV causes^b^	0.73 (0.59–0.89)	

Adverse events^c^	HR (95% CI)	
Serious urinary tract infection	0.94 (0.64–1.37)	
Serious hyperkalemia	0.83 (0.63–1.09)	
Serious acute kidney injury	0.78 (0.60–1.00)	
Serious dehydration	1.25 (0.73–2.14)	
Severe hypoglycemia	1.00 (0.73–1.37)	

^a^The analysis of hospitalizations for any cause included the first and all subsequent events, so only the rates are shown; 1611 hospitalizations occurred among 960 participants in the empagliflozin group, and 1895 hospitalizations occurred among 1035 participants in the placebo group

^b^End-stage kidney disease was defined as the initiation of maintenance dialysis or receipt of a kidney transplant

^c^Selected adverse events

**Table 3:** Key information of the SURMOUNT-1 trial [30]

	Tirzepatide, 5 mg	Tirzepatide, 10 mg	Tirzepatide, 15 mg	Placebo
	least-squares mean (95% CI)			
**Co-primary endpoints**^a^				
Change in body weight (in %)	− 15.0	− 19.5	− 20.9	− 3.1
Difference from placebo in percentage change in body weight (in %)	− 11.9	− 16.4	− 17.8	–
Weight reduction of 5% or more at week 72 (% of participants)	85.1	88.9	90.9	34.5
**Key secondary endpoints**^a^				
Weight reduction of 10% or more at week 72 (% of participants)	68.5	78.1	83.5	18.8
Weight reduction of 15% or more at week 72 (% of participants)	48.0	66.6	70.6	8.8
Weight reduction of 20% or more at week 72 (% of participants)	30.0	50.1	56.7	3.1
Change in waist circumference (in cm)	− 14.0	− 17.7	− 18.5	− 4.0
Difference from placebo in change in waist circumference (in cm)	− 10.1	− 13.8	− 14.5	–
**Gastrointestinal (GI)-related adverse events (occurring in at least 5% of the participants)**				
Nausea (Event rate in %)	24.6	33.3	31.0	9.5
Diarrhea (Event rate in %)	18.7	21.2	23.0	7.3
Constipation (Event rate in %)	16.8	17.1	11.7	5.8
Dyspepsia (Event rate in %)	8.9	9.7	11.3	4.2
Vomiting (Event rate in %)	8.3	10.7	12.2	1.7

Data shown as Treatment-Regimen-Estimands

^a^The primary and key secondary end points were tested under a type 1 error–control procedure, and all comparisons with placebo were significant at p < 0.001

### SGLT2 inhibitors

#### DELIVER

The DELIVER trial [28] evaluated the effects of dapagliflozin (10 mg/daily) in 6263 participants with heart failure with preserved ejection fraction (HFpEF). Participants were eligible if they were at least 40 years of age, had stabilized HF with or without T2D, a left ventricular ejection fraction greater than 40%, evidence of structural heart disease, and elevated natriuretic peptide levels [28]. The primary endpoint was the occurrence of worsening HF or CV death and was assessed in a time-to-event analysis using a Cox proportional hazards model. Worsening HF was defined as an unplanned hospitalization for HF or an urgent visit for HF. Several secondary and safety outcomes were prespecified by the investigators, including the total number of events of worsening HF, CV death, and serious adverse events. Additional prespecified outcomes are shown in Table 1 [28].

During a median follow-up of 2.3 years, dapagliflozin demonstrated a significant improvement in the primary composite outcome with an 18% reduction in the combined relative risk of worsening HF or CV death (hazard ratio (HR) 0.82 [95% confidence interval (CI) 0.73–0.92]; p < 0.001). The primary outcome event was observed in 16.4% (n = 512) of the dapagliflozin group and among 19.5% (n = 610) of the placebo group. Similar results were observed in participants with an ejection fraction of less than 60% compared to those of the overall population (HR 0.83 [95% CI 0.73–0.95]; p = 0.009). Furthermore, results were similar in pre-specified subgroups, including participants with or without diabetes [28]. When evaluating individual components of the primary outcome, a reduction in the rate of hospitalization for HF or urgent visit for HF (HR 0.79 [95% CI 0.69–0.91]) and CV death (HR 0.88 [95% CI 0.74–1.05]) was observed in the dapagliflozin group compared with the placebo group.

Secondary outcomes of interest included a significant reduction in the total number of a composite of worsening HF events and CV death with dapagliflozin use, with 815 such events occurring in the dapagliflozin group and 1057 occurring in the placebo group (HR 0.77 [95% CI 0.67–0.89]; p < 0.001). In the safety analyses, serious adverse events, including death, occurred in 43.5% (n = 1361) of the dapagliflozin group and in 45.5% (n = 1423) of the placebo group. The investigators also noted that 5.8% of the dapagliflozin group and 5.8% of the placebo group discontinued treatment due to adverse events [28].

#### EMPA-KIDNEY

The EMPA-KIDNEY trial [29] evaluated the efficacy and safety of empagliflozin (10 mg/daily) on the progression of kidney disease and CVD in 6609 participants with CKD. Eligible participants were individuals with or without diabetes, with an eGFR ≥ 20 to < 45 mL/min/1.73 m^2^, regardless of the level of albuminuria, or with an eGFR ≥ 45 to < 90 mL/min/1.73 m^2^ with a UACR of at least 200 mg/g (20 mg/mmol) [39].

The primary outcome was a composite of progression of kidney disease and death from CV causes, with kidney disease progression being defined as kidney failure, sustained decrease in eGFR to < 10 mL/min/1.73 m^2^, sustained decrease in eGFR of ≥ 40% from baseline, or kidney death. The key secondary outcomes were hospitalization for HF or death from CV causes, hospitalization for any cause and death for any cause. A Cox proportional hazards model was used for time-to-event analyses of empagliflozin versus placebo. Several safety outcomes and adverse events were analyzed by the investigators [29], a selected list of which is shown in (Table 2).

The trial was stopped after a median follow-up of 2.0 years due to positive efficacy, which met the pre-specified threshold for early termination. Study analysis demonstrated that kidney disease progression or CV death occurred in 13.1% (n = 432) of participants in the empagliflozin group and 16.9% (n = 558) of participants in the placebo group (HR 0.72; CI 0.64–0.82; p < 0.001) [29]. The rate of hospitalization for any cause was also lower in the empagliflozin group than in the placebo group (24.8 vs. 29.2 hospitalizations per 100 patient-years; HR 0.86; CI 0.78–0.95; p = 0.003) [29].

However, the difference in the rate of hospitalization for HF or death from CV causes between the empagliflozin (4.0%) and placebo (4.6%) groups was not found to be significant (HR 0.84; CI 0.67–1.07; p = 0.15) [29]. The same observation was made regarding death from any cause (4.5% in the empagliflozin group vs. 5.1% in the placebo group; HR 0.87; CI 0.70–1.08; p = 0.21), and the occurrence of serious adverse events, the incidences of serious urinary tract infection, hyperkalemia, acute kidney injury, dehydration, severe hypoglycemia were broadly similar in the two groups (Table 2). This was the first kidney trial with an SGLT2i that included people with normal albumin excretion, and in this subgroup with a lower event rate, there was no significant effect on the primary endpoint, but a significant reduction in the rate of decline in kidney function comparing the empagliflozin group [0.11 (0.17) ml/min/1.73 m^2^/year] to placebo [0.89 (0.16) ml/min/1.73 m^2^/year] [29].

Overall, empagliflozin reduced the relative risk of kidney disease progression or death from CV causes by 28% and the risk of hospitalization for any cause by 14% in individuals with or without diabetes and with an eGFR ≥ 20 mL/min/1.73 m^2^ [29].

### GLP-1 receptor agonists

Following the AMPLITUDE-O CVOT [15], the randomized, placebo-controlled trial AMPLITUDE-M analyzed the glycemic efficacy and safety of once-weekly efpeglenatide (2, 4 or 6 mg) in T2D [42]. Safety assessments included hypoglycemia, severe gastrointestinal events and selected CV events [42]. Gastrointestinal events were the most common class of adverse events reported and the main cause of discontinuation of efpeglenatide treatment, while the incidence of hypoglycemia was low and no deaths were reported [42].

### Weight loss trials in people with obesity and overweight

#### Surmount-1

The SURMOUNT-1 trial [30] evaluated the efficacy and safety of tirzepatide, a GIP and GLP-1 RA versus placebo in adults with overweight or obesity and a BMI of 30 kg/m^2^ or greater or 27 kg/m^2^ plus at least one weight-related complication (e.g., hypertension, dyslipidemia, obstructive sleep apnea, or CVD), excluding diabetes. The trial enrolled 2539 participants with a mean age of 45 ± 13 years, a mean body weight of 105 kg, and a mean BMI of 38. A total of 95% of participants had a BMI of > 30 at baseline. Participants were randomized in 1:1:1:1 fashion to tirzepatide 5 mg, 10 mg, or 15 mg, or placebo administered subcutaneously once a week for 72 weeks in addition to the lifestyle intervention. The starting dose (2.5 mg) was gradually increased at 4 week intervals. Common key exclusion criteria were diabetes, a change in body weight of more than 5 kg within 90 days prior to screening, previous or planned surgical treatment for obesity, and treatment with a weight loss medication within 90 days prior to screening [30].

The co-primary endpoints were the percentage change in body weight from baseline to week 72 and the percentage of participants achieving body weight reductions of a 5% or more at week 72 compared to placebo. Key secondary endpoints included weight reductions of 10% or greater, 15% or greater, and 20% or greater at week 72; the change in body weight from baseline to week 20; and the change from baseline to week 72 in waist circumference, systolic blood pressure, fasting insulin, and lipid levels [30].

For the co-primary endpoint of percentage change in body weight, the mean change in body weight at week 72 was − 15.0% (95% CI − 15.9 to − 14.2) with tirzepatide 5 mg, − 19.5% (95% CI − 20. 4 to − 18.5) with tirzepatide 10 mg and − 20.9% (95% CI − 21.8 to − 19.9) with tirzepatide 15 mg compared to -3.1% (95% CI − 4.3 to − 1.9) with placebo (p < 0.001 for all comparisons). The co-primary endpoint of body weight loss of 5% or greater was achieved by 85% (95% CI 82–89) of those treated with tirzepatide 5 mg, 89% (95% CI 86–92) of participants treated with tirzepatide 10 mg, and 91% (95% CI 88–94) of participants treated with tirzepatide 15 mg compared to 35% (95% CI 30–39) of participants treated with placebo (p < 0.001 for all comparisons). In addition, 50% (95% CI 46–54) of the tirzepatide 10 mg group and 57% (95% CI 53–61) of the tirzepatide 15 mg group had a reduction in body weight of 20% or more.

This level of body weight loss was achieved by only 3% (95% CI 1–5) of participants. In addition, at week 72, 95% of participants with prediabetes at baseline in the tirzepatide group had returned to normoglycemia. Among secondary endpoints, tirzepatide use was associated with favorable changes in waist circumference, systolic and diastolic blood pressure, fasting insulin and lipid levels. When assessing safety outcomes, 79–82% of participants receiving tirzepatide reported at least one adverse event during the treatment period compared to 72% in the placebo group. The most common adverse events were gastrointestinal and generally mild to moderate in severity, usually occurring during the dose-escalation period. Nausea, diarrhea, and constipation were the most common adverse events (Table 3).

## Key topics discussed during the 8th CVOT Summit

### Key aspects of the ADA-EASD consensus report 2022

The ADA and EASD issued a consensus report on the management of hyperglycemia in T2D in 2022 [35]. The joint statement focuses on a holistic, person-centered approach to the management of T2D and considers cardiorenal protection as well as glycemic, body weight, and CV risk management as components of care. In principle, the choice of glucose-lowering agents should be guided by the individual profile and the presence of comorbidities such as obesity, CVD, HF, CKD, and non-alcoholic fatty liver disease (NAFLD). In people with HF, CKD, established CVD, or multiple risk factors for CVD, the decision to use SGLT2is or GLP-1 RAs with proven benefit should be independent of baseline HbA1c or background metformin use. Especially in people with HF and in people with CKD and eGFR ≥ 20 mL/min/1.73 m^2^, SGLT2 inhibitors with proven benefit are now preferred and should also be used to reduce MACE, HF and to improve kidney outcomes in people with established CVD [32].

### Network meta-analysis of RCTs as evidence support for updating clinical practice guidelines

Rapid and accurate systematic review of clinical data and reliable information is needed to update clinical practice guidelines (CPG) as new research evidence emerges. To address this challenge, the WHO and other organizations and groups introduced the “living guideline”, a mechanism for continuous evidence monitoring of selected CPG recommendations [43]. The “living guidelines” concept gained notoriety during the COVID-19 pandemic [44]. Eventually, other medicine research areas, where research evidence is emerging rapidly, are adapting “living guidelines” methods.

In 2019, an interdisciplinary expert’s panel (Taskforce of the Guideline Workshop) convened to develop strategies to improve speed, efficiency, and effectiveness of guideline development processes as applied to diabetes, CVD, and kidney diseases [45]. Following that, a clinical practice guideline for the use of SGLT2is and GLP-1 RAs in the management of T2D in very high risk individuals, with CVD and CKD as comorbidities, was developed using the MAGICapp platform [46]. The guideline recommendations are based on a well conducted and rigorous systematic review and network meta-analysis of RCTs with these medicines [47]. The purpose of this research work is to collect evidence-based support for updating the recommendations in the guideline regarding the role of different medications in clinical practice including newer medications like a nonsteroidal MRA.

### Implementation of guidelines and optimization of standards of care

Clinical practice guidelines and standards of care have been updated in recent years based on evidence from major CVOTs. However, implementation in clinical practice is low and improving outcomes remains a challenge. Fewer than one in 20 people with T2D and ASCVD are receiving all guideline-recommended therapies (high-intensity statin, angiotensin-converting enzyme inhibitors (ACEi) or angiotensin receptor blockers (ARB), SGLT2i or GLP-1 RA) [48]. This delay has been attributed to the existence of therapeutic inertia among clinicians, and barriers among patients and the health care system [49]. These obstacles should be addressed with appropriate strategies and interventions. To evaluate the efficacy of a clinic- and individual-level educational intervention to improve knowledge on and thereby uptake of evidence-based therapies and management of T2D and CVD, a prospective trial, COORDINATE-Diabetes [49], was launched across the USA in 2022. The trial is ongoing, and its rationale and design have recently been published [49]. Results are expected in 2023.

To raise awareness and promote the implementation of guidelines for heart and kidney protection, a global initiative, Guardians for Health, has recently been launched to support healthcare professionals in providing state-of-the-art care (https://www.guardiansforhealth.com).

### Towards reclassification of T2D

T2D is recognized for its heterogeneity and has been reclassified in recent years into subgroups based on age and Hb1Ac at diagnosis, glutamic decarboxylase antibodies, BMI, and homeostatic model assessment estimates of β-cell function and insulin resistance [50]. The novel T2D subgroups: severe insulin-deficient diabetes (SIDD), severe insulin-resistant diabetes (SIRD), mild obesity-related diabetes (MOD), and mild age-related diabetes (MARD), have been found to be predisposed to different risks of developing CVD, CKD, or diabetic retinopathy [50]. SIDD has been found to be prone to retinopathy and polyneuropathy [50, 51], whereas SIRD has been found to be prone to CKD and NAFLD [50, 51]. A recent retrospective analysis of data from the ORIGIN trial confirmed these observations [52]. Large differences between subgroups have been attributed to genetic variation and epigenetic differences [50, 53]. Clustering methods have also been performed in individuals at increased risk of T2D (prediabetes), identifying six clusters with different propensities to develop diabetes and diabetes-related complications [54]. Through the use of new classifications, it may be possible to tailor treatment to people at highest risk and potentially develop therapeutic interventions for those who will benefit the most or respond the best.

### Glycemic management, continuous glucose monitoring (CGM), and time in range (TIR) in T2D

T2D is considered a complex and progressive disease characterized by glycemic disturbances, including both sustained chronic hyperglycemia and acute glucose fluctuations. Typically, HbA1C has been used as a marker to assess glycemic status and even to guide therapy decisions, but this has not resulted in optimal glycemic control [55]. HbA1c reflects the average glucose over the last 2–3 months, and it is a valuable marker to assess the risk of microvascular complications or even mortality from early on [56]. Unlike the HbA1c measurement, the use of continuous glucose monitoring (CGM) allows the direct observation of glycemic excursions and daily profiles, which can inform on immediate therapy decisions and/or lifestyle modifications. CGM generates a huge amount of data that is difficult to manage. Therefore, data visualization has been standardized to a one-page ambulatory glucose profile (AGP) report [57]. In the MOBILE study, significantly lower HbA1c levels were observed in people with T2D on basal insulin (no prandial insulin) after using a CGM system for 8 months, when compared to blood glucose meter monitoring. [58, 59].

As of 2022, the ADA recommends CGM for both rapid-acting and long-acting (basal) insulin-treated adults [60]. Ultimately, the number of CGM users will increase, but better health outcomes can only be achieved if both users and physicians are able to interpret the AGP and CGM data and to make appropriate lifestyle and medication adjustments. The nine- and five-step AGP interpretation procedures described in the clinical guidelines [61, 62] are not considered simple and clear enough. Therefore, a three-step analysis approach to facilitate the interpretation of CGM data and the AGP report has recently been proposed [63] and a follow-up model for clinician CGM guided management (CCGM) has been developed and presented at the CVOT Summit (Bergenstal, CVOT 2022, personal communication).

### Combination therapies and developments of new insulin preparations

#### Combination therapies in T2D

Insulin is an effective glucose-lowering agent, but there are also other glucose-lowering agents that can be used for initial treatment of T2D. In particular, GLP-1 RAs should be considered for use before initiating insulin therapy in the absence of contraindications [35] because of the reduced injection burden, reduced risk of hypoglycemia [64], and potential for body weight loss [35]. However, insulin may be preferred for glucose-lowering in the setting of severe hyperglycemia (HbA1c > 10% (86 mmol/mol), especially when associated with body weight loss or ketonuria/ketosis and with acute glycemic dysregulation (e.g., during hospitalization for myocardial infarction, surgery or acute illness), in underweight people or when the diagnosis of T1D is suspected [35].

If glycemic targets are not met under other pharmacotherapy, the addition of insulin should be considered [35]. Recently, the SoliMix trial of once-daily iGlarLixi, a combination of insulin glargine and lixisenatide (GLP-1 RA), reported benefits in terms of better glycemic control, body weight control, and reduced hypoglycemia versus twice daily biphasic insulin in people with suboptimal controlled T2D [65].

#### Inhaled insulin

As an alternative to subcutaneous insulins, an inhaled ultra-rapid-acting insulin with an onset of action of approximately 12 min and a shorter duration of action (typically ≤ 3 h) has been developed and is available in the USA for use in both type 1 diabetes (T1D) and T2D [66]. The concept behind this technological innovation is to take advantage of the large pulmonary surface area, which allows insulin to be delivered into the bloodstream in approximately 1 min [66]. In a recent meta-analysis of insulin RCTs in T1D, inhaled insulin showed less weight gain and fewer hypoglycemic shifts, with a similar effect on the blood glucose level when compared to other insulin types [67]. In T2D, TI was found to improve HbA1c and TIR, with low rates of hypoglycemia [66]. Cough is the most common non-hypoglycemic adverse event associated with inhaled insulin. It has been found to be generally mild and dry. It has also been reported to occur within 10 min of inhalation, to be transient, and to decrease with continued use. Overall, 94% of cough episodes are characterized as intermittent or single defined episodes [66].

#### NAFLD/NASH trials

NAFLD and non-alcoholic steatohepatitis (NASH) affect 55.5% and 37.3% of individuals with T2D, respectively [68], and are associated with poor clinical outcomes including mortality, CV disease and CKD [69]. Lessons learned from CVOTs trials are helping to shape trials in NAFLD and NASH, and several glucose-lowering therapies are being studied in this area. However, trial recruitment in NASH remains an obstacle due to lack of access to patients and sites, inefficiencies in patient enrollment, current lack of non-invasive validated biomarkers of NASH and lack of access to biopsy samples. ProSciento, a clinical research organization specializing in metabolic diseases, has been successful in overcoming these barriers with an Institutional Review Board-approved diagnostic clinical research protocol (NASH PASS^®^), patient registry, and real-time and longitudinal biobank (www.prosciento.com). The approach leverages the NASH PASS^®^/PRO PASS platforms to provide the expertise and infrastructure necessary to design and conduct interventional clinical trials in NASH and fibrotic phenotypes.

#### CKD management

Kidney Disease: Improving Global Outcomes (KDIGO) has recently published clinical practice guidelines for diabetes management in CKD [70], recommending SGLT2is as first-line drug therapy and considering nonsteroidal MRA (finerenone) for additional risk-based therapy in individuals with persistent albuminuria despite use of ACE or ARB [70]. GLP-1 RAs are not currently indicated to improve kidney outcomes, although there is accumulating evidence from CVOTs of a kidney-and cardioprotective effect in people with T2D and CKD [71]. However, since GLP-1 RAs effectively reduce HbA1c and CKD is associated with CVD, the guideline recognizes the potential of GLP-1 RAs as adjuncts to metformin and SGLT2i. Both the ADA and the KDIGO recommend annual screening for CKD in people with diabetes by measuring both UACR and eGFR [72]. The first dedicated kidney trial to evaluate a GLP-1 RA will be FLOW with semaglutide in T2D with CKD. Report expected in 2024 [73].

#### Body weight loss and obesity management

Recent evidence suggests that body weight loss of 5–15% should be a primary goal for people with T2D and obesity [74], as it is associated with improvements in blood glucose control, risk factors for cardiometabolic disease, and quality of life [74]. In addition to diet and behavioral therapy, pharmacotherapy with GLP-1 RAs is also an indication for body weight management in people with T2D [35]. Recently, tirzepatide, a GIP/GLP-1 RA that significantly reduces HbA1c levels in people with T2D [75], has also been shown to have remarkable effects on body weight loss in people with and without diabetes [30]. However, tirzepatide is not yet approved by regulatory authorities for body weight management. It is approved in the USA, Europe and the UAE in 2022 to improve glycemic control in adults with T2D in addition to a healthy diet and exercise.

#### Real-world evidence (RWE) and future clinical trial design

The FDA defines real-world data (RWD) as “data related to the patient’s health status and/or health care delivery that are routinely collected from a variety of sources.” These include electronic health records, medical claims and billing data, data from product and disease registries, data collected from patients, and data from sources that can provide information about health status [76]. After analysis, RWD is translated to real-world evidence (RWE) about treatment, risks, and safety of medicinal products and procedures. Eventually, RWE is evaluated by regulatory authorities who consider it during the decision-making processes on patient’s access to therapies [77, 78]. Furthermore, RWE influences the methodology and study design of new RCTs and non-interventional studies by generating hypotheses to be tested in RCTs, assessing the feasibility of a trial under defined inclusion and exclusion criteria in a geographic area, informing about prior probability distributions, or even identifying prognostic indicators or baseline patient characteristics [76].

RWE studies can also complement RCTs by providing valuable information on the effectiveness and safety of RCT-validated drugs for individuals with multiple comorbidities and on multiple drug therapy, who are typically excluded from trials. [79]. One such example is CVD-REAL, which confirmed reductions in risk of HF hospitalization and all-cause mortality in real-world individuals with and without CVD taking SGLT2is [80, 81]. RWE studies are also important to confirm and extend CVOT results in the real world, as shown by the analysis of the real-world US claims database on the risk of stroke in individuals with T2D receiving semaglutide (GLP-1 RA) or a DPP-4i [82]. According to the study, individuals with T2D initiating semaglutide have a lower risk of stroke than those initiating a DPP-4i (HR 0.63 [95% CI 0.41, 0.95]; p = 0.029), with the effect being more pronounced in individuals with T2D and ASCVD (HR 0.45 [0.24, 0.86]; p = 0.015) [82].

Worldwide, RWD are being collected through globally established RWE programs. The DOPPS program (https://www.dopps.org), has followed more than 120,000 individuals on hemodialysis and peritoneal dialysis as well as people with CKD in over 20 countries since 1996 and supports observational studies to identify best practices in nephrology. The DOPPS database is included in the DISCOVER CKD program [83], a global observatory hybrid study of the epidemiology and management of CKD integrating data from retrospective and prospective cohorts. The study is ongoing and will collect information from both routine clinical visits and patient-related outcomes on CKD management and CKD comorbidities, in particularly diabetes and cardiovascular complications [83].

## Conclusion

The 8th CVOT Summit on Cardiometabolic, Kidney and Glycemic provided an interactive and multidisciplinary platform to discuss key results from recently published trials. The summit covered two recent outcome trials with SGLT2i (DELIVER and EMPA-KIDNEY) and one chronic weight management trial with GIP and GLP-1 RA (SURMOUNT-1). The meeting discussed new data, insights, and strategies for both specialists and primary care physicians for the management of diabetes, obesity, HF, CVD, and CKD. In addition, key topics such as guideline implementation and optimization of standards of care, network meta-analysis, combination therapies, development of new insulin formulations, and real-world evidence for future clinical trial design were discussed. In-depth discussions and presentations of upcoming CV, kidney, glycemic, and obesity trials will continue at the 9th CVOT Summit, which will be held virtually on November 23–24, 2023 (www.cvot.org).

## Data Availability

Data sharing not applicable to this article as no datasets were generated during the current study.

## Abbreviations

ACEi: Angiotensin-converting-enzyme inhibitor
ADA: American Diabetes Association
AGP: Ambulatory glucose profile
ARB: Angiotensin-receptor blocker
ARNI: Angiotensin receptor-neprilysin inhibitor
ASCVD: Atherosclerotic cardiovascular disease
AWMF: Arbeitsgemeinschaft der Wissenschaftlichen Medizinischen Fachgesellschaften
BMI: Body mass index
CGM: Continuous glucose monitoring
CI: Confidence interval
CKD: Chronic kidney disease
CV: Cardiovascular
CVD: Cardiovascular disease
CVOT: Cardiovascular outcome trial
DKD: Diabetic kidney disease
DPP-4i: Dipeptidylpeptidase-4 inhibitor
eGFR: Estimated glomerular filtration rate
EMA: European Medicines Agency
ESC: European Society of Cardiology
FDA: U.S. Food and Drug Administration
GIP: Glucose-dependent insulinotropic polypeptide
GLP-1 RA: Glucagon-like peptide-1 receptor agonist
HbA1c: Glycated hemoglobin 1Ac
HF: Heart failure
HFmrEF: Heart failure with mildly reduced ejection fraction
HFpEF: Heart failure with preserved ejection fraction
HFrEF: Heart failure with reduced ejection fraction
HHF: Hospitalization for heart failure
HR: Hazard ratio
IDF: International Diabetes Federation
KCCQ: Kansas city cardiomyopathy questionnaire
LVEF: Left ventricular ejection fraction
MACE: Major adverse cardiovascular event
MI: Myocardial infarction
MRA: Mineralocorticoid receptor antagonist
NAFLD: Non-alcoholic fatty liver disease
NASH: Non-alcoholic steatohepatitis
NICE: National Institute for Health and Care Excellence
NYHA: New York Heart Association
RAS: Renin-angiotensin system
RCT: Randomized controlled trial
RWD: Real-world data
RWE: Real-world evidence
SC: Subcutaneous
SGLT2i: Sodium-glucose cotransporter-2 inhibitor
T1D: Type 1 diabetes mellitus
T2D: Type 2 diabetes mellitus
TIR: Time in range
UACR: Urine albumin-to-creatinine ratio
WHO: World Health Organization
